# Antibiotic Prescribing Practices in a Tertiary Care Teaching Hospital: A Retrospective Cross-Sectional Analysis

**DOI:** 10.7759/cureus.73092

**Published:** 2024-11-05

**Authors:** Usman Zafar, Ashir Iqbal, Abdul Basit, Muhammad S Ahmed, Hafiz Muddassir Imtiaz, Ferwa Asif, Moiz N Butt, Ahmed Faraz, Sundas N Butt, Muhammad A Awan

**Affiliations:** 1 Department of Medicine, Dr. Akbar Niazi Teaching Hospital, Islamabad, PAK; 2 Hospital Administration, Alkhidmat Raazi Hospital, Rawalpindi, PAK; 3 Internal Medicine, Alkhidmat Raazi Hospital, Rawalpindi, PAK; 4 Family Medicine, Alkhidmat Raazi Hospital, Rawalpindi, PAK; 5 General Medicine, Dr. Akbar Niazi Teaching Hospital, Islamabad, PAK; 6 Cardiothoracic Surgery, Guy's and St Thomas' NHS Foundation Trust, London, GBR; 7 Emergency Medicine, Cambridge University Hospitals, Cambridge, GBR; 8 General Surgery, Dr. Akbar Niazi Teaching Hospital, Islamabad, PAK

**Keywords:** antibiotic prescribing practices, antibiotic stewardship programs, antimicrobial resistance, culture sensitivity testing, evidence of infection, rational use of antibiotics

## Abstract

Background

Antibiotics are among the most frequently prescribed medications in hospitals, yet a significant portion of their use is inappropriate, contributing to the growing global threat of antimicrobial resistance (AMR). In Pakistan, AMR has reached alarming levels with the rise of multi- and extensively drug-resistant bacteria. This study evaluates antibiotic prescribing practices and the use of culture sensitivity (CS) testing to assess the appropriateness of antibiotic therapy.

Methods

This cross-sectional retrospective study was conducted in a 500-bed tertiary care teaching hospital in Islamabad, Pakistan, analyzing inpatient records from 2020 to 2022. From over 5000 patient files, 1012 met the inclusion criteria and were reviewed. The study assessed the rationality of antibiotic prescriptions based on the evidence of infection (EoI), clinical parameters, and the presence or absence of CS testing. Statistical analyses, including Chi-square tests, logistic regression, and Cox proportional hazards regression, were applied to determine the association between antibiotic use and patient outcomes.

Results

Among the 1,012 patients analyzed, 91.8% (n = 929) received one or more antibiotics. However, 30% (n = 274) of these prescriptions were issued without any documented EoI. Only 17.5% of patients underwent CS testing. Patients exposed to five or more antibiotics had a 2.5-fold increased risk of ICU mortality (HR = 2.50, p < 0.001). A positive correlation (r = 0.42, p < 0.001) was found between the number of antibiotics prescribed and the length of hospitalization.

Conclusion

The findings of this study reveal a high rate of inappropriate antibiotics prescribed without EoI and CS testing. The results emphasize the urgent need for comprehensive Antibiotic Stewardship Programs (ASPs) in hospital settings, particularly focusing on mandatory CS testing protocols. By reducing irrational antibiotic use, these initiatives can significantly mitigate the rise of AMR globally, especially in resource-limited settings. Implementing ASPs not only optimizes antibiotic use but also aligns with the WHO recommendations, demonstrating the effectiveness of multifaceted interventions in minimizing resistance and improving patient outcomes.

## Introduction

Antimicrobial resistance (AMR) has reached critical levels globally, with low- and middle-income countries, including Pakistan, being particularly vulnerable due to weaker regulatory frameworks [1]. The 2017 WHO Global Action Plan on AMR highlighted the need for improved antibiotic stewardship worldwide, particularly in countries where over-the-counter availability of antibiotics is common. In Pakistan, previous attempts to curb antibiotic misuse have been limited in scope, with challenges in the implementation of national guidelines, such as the National Action Plan on AMR. Studies have shown that the irrational and excessive use of antibiotics in Pakistani hospitals is a major driver of AMR [2,3]. A 2020 study by Khan et al. [2] emphasized the role of limited diagnostic facilities and unregulated prescription practices as major barriers to effective antibiotic use and documented the high prevalence of extended-spectrum β-lactamase (ESBL)-producing Enterobacteriaceae in Pakistan.

The WHO’s first global report on antibiotic resistance (2014) declared AMR as an alarming public health threat requiring global efforts [4]. Both worldwide, and especially in underdeveloped countries, the prevalence of AMR is a threat of great magnitude. About two-thirds of antibiotic resistance arises from the Asia region, with South Asia, given its large population, considered the epicenter of resistant microorganisms [1].

In Pakistan, the prevalence of antibiotic resistance is a significant hazard to the healthcare delivery system [2]. In recent years, the emergence of multidrug-resistant (MDR) and extensively drug-resistant (XDR) organisms has raised serious concerns [3]. Various studies have revealed that irrational use, overprescribing, lack of training, self-medication, incomplete dosage by patients, and the absence of culture sensitivity (CS) tests are contributing causes of antibiotic resistance [5,6]. Pakistan's National Action Plan (2017) on AMR, while aligned with WHO's global strategies, has struggled with implementation due to insufficient logistical support and local engagement [7,8]. In Pakistan, cephalosporins are commonly prescribed antibiotics in the hospital setting for respiratory tract infections, urinary tract infections, and surgical prophylaxis. The choice of these antibiotics reflects a regional preference due to their broad-spectrum activity and affordability, despite the growing concerns of resistance [9].

Several physician-related factors are implicated in inappropriate and unnecessary antibiotic prescriptions, including the perceived compulsion to prescribe antibiotics from the patient or their parents (in the case of children), inability to differentiate minor self-limiting viral ailments from severe bacterial infections, hastened patient consultations, a lack of up-to-date knowledge of guidelines, and managing a larger patient load [10-12].

Studies aimed at analyzing the rise in antibiotic resistance have consistently recommended reducing the irrational use of antibiotics as a key preventive measure [13]. To ensure the appropriate use of antibiotics and minimize harm, antibiotic stewardship programs (ASPs) have been developed [14,15]. This development was important, as earlier studies showed that targeted interventions focusing solely on physician behavior and traditional education resulted in little to no improvement in prescribing practices [16,17]. Multifaceted complex interventions involving physician behavior, patient concerns, and public awareness are more effective at reducing inappropriate antibiotic use for various conditions. The implementation of antimicrobial stewardship interventions has been shown to improve the appropriateness of broad-spectrum antibiotic usage [18].

The primary objective of this study is to analyze antibiotic prescribing patterns among hospitalized patients, emphasizing targeted solutions for reducing excessive antibiotic use, including implementing ASPs and mandatory CS testing. This study also aims to provide actionable recommendations for improving antibiotic stewardship practices within hospitals.

## Materials and methods

Study design, setting, and study population

The study design is a retrospective cross-sectional analysis of electronic patient records from 2020 to 2022, obtained from a tertiary care teaching hospital in Islamabad, Pakistan. From an initial dataset of 5,343 patient files, 1,012 met the inclusion criteria. The primary inclusion criterion was a minimum hospital stay of four days, which ensured sufficient time for antibiotic administration and observation of treatment outcomes. Exclusion criteria included daycare patients and cases with an inpatient stay of less than four days. All patients included in this study were admitted through a government-funded insurance program called the "Sehat Sahulat Program."

**Figure 1:**
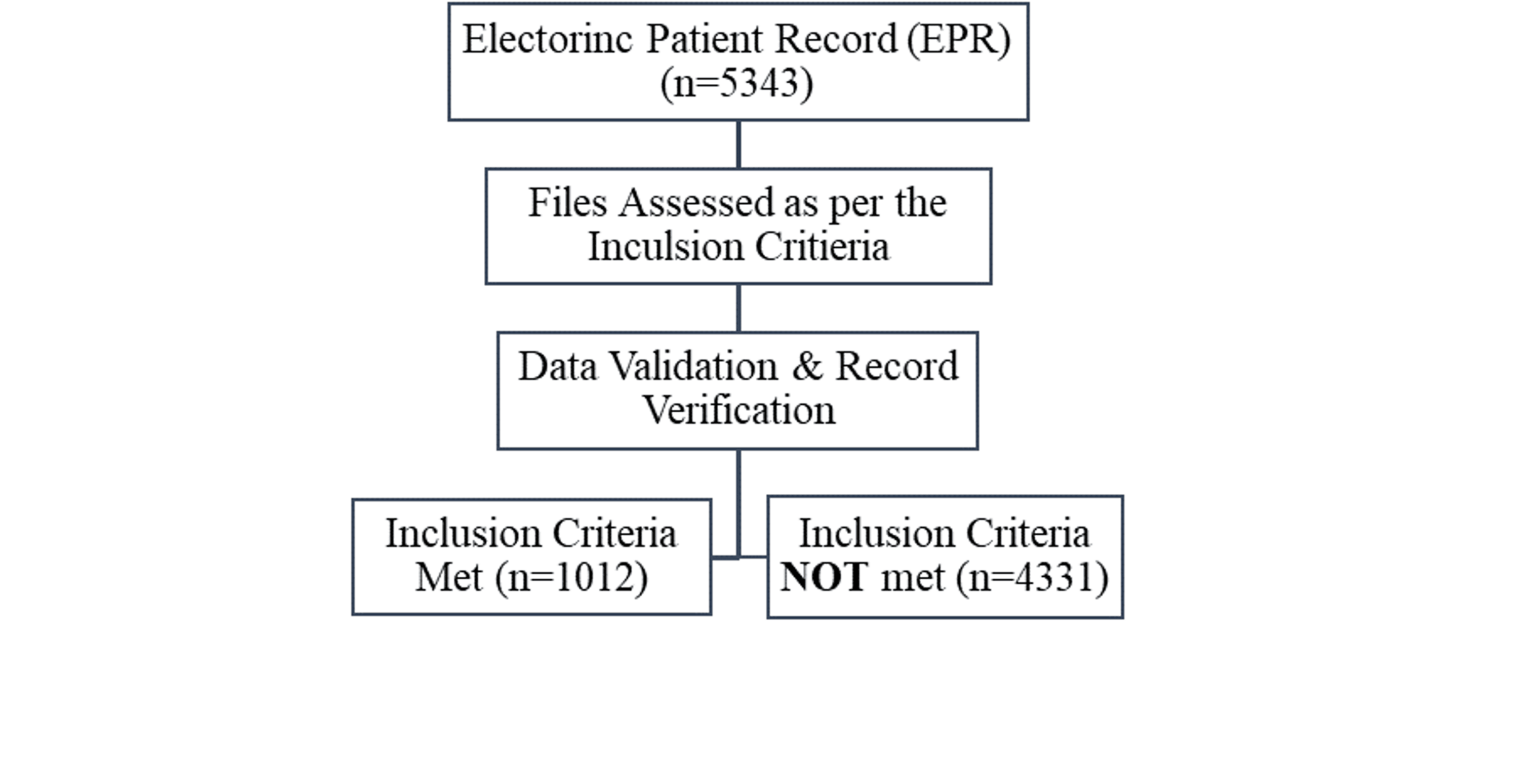
Summary of data recruitment

Due to the hospital's data encryption and entry process, the records were provided in phased periods. The phased data collection over the year and the large volume of records resulted in time gaps in data collection and analysis. However, rigorous screening and validation ensured the integrity of the dataset used for the final analysis. Only records that met the inclusion criteria of a minimum hospital stay of four days were included in the analysis. Additionally, data entries were cross-verified with other hospital records and/or patient summaries to ensure consistency. Figure 1 represents the data collection and analysis process.

Inclusion and exclusion criteria

Patients with a minimum hospital stay of four days were deemed eligible for inclusion in the study. All daycare patients, one-day admission cases, and those whose length of inpatient stay was less than four days were excluded.

Data collection

The electronic database record included details regarding admission and discharge, medicine administration records (MAR), and laboratory investigations. The abovementioned parameters were recorded into a self-designed standardized perfoma and files were analyzed. The data form included the following elements: demographics, admitting and discharging diagnosis, comorbids, evidence of infection (EoI), laboratory investigations, and antibiotic administration record sheet (containing details of dose, route of administration, frequency, and duration of therapy).

Rationality of antibiotic therapy

EoI was defined through multiple parameters: documented fever spikes (above 38°C), elevated total leukocyte counts (>12,000 cells/mm³), positive radiological evidence (such as chest X-rays), and confirmed culture positivity (urine or blood cultures). The combination of clinical signs and laboratory data minimized potential bias in assessing the appropriateness of antibiotic therapy. Records were cross-verified through multiple sources, including hospital summaries and lab reports, to ensure the accuracy of data.

Ethical consideration

Ethical approval for this study was obtained from the Institutional Review Board (IRB) of the hospital. As this study utilized retrospective patient data, informed consent was waived by the IRB, adhering to local regulations and ethical guidelines. All data were anonymized before analysis to ensure patient confidentiality, with strict adherence to the hospital’s data protection protocols.

Statistical analysis

Descriptive and advanced statistics were applied to the data collected using MS Excel (Microsoft Corporation, Redmond, Washington, United States) and IBM SPSS Statistics for Windows, Version 25 (Released 2017; IBM Corp., Armonk, New York, United States).

## Results

The total number of files analyzed out of 5343 was 1012, meeting the inclusion criteria. The mean age of the pediatric population was 3.96 (± 3.7 SD), and for the adults, the mean age was 49.31 (±17.47 SD), with a male-to-female ratio of approximately 1:1. An independent t-test showed no significant difference in age distribution between males and females (p = 0.07). The mean length of hospitalization was 6.75 (±2.89 SD) days. Analysis of variance (ANOVA) tests revealed significant differences in the length of hospitalization across departments (F = 5.91, p < 0.01), with patients in General Medicine having significantly longer hospital stays compared to those in Surgery (p < 0.05). A Chi-square test for age distribution across different departments showed significant differences (χ² = 38.72, p < 0.001), indicating that older patients were more likely to be admitted to General Medicine and Nephrology departments. The age distribution of hospitalized patients is shown in Table 1.

**Table 1:** Age distribution χ²: Chi-square test; F: ANOVA; OR: odds ratio; ANOVA: analysis of variance Significance level: p < 0.05

Age category (years)	Frequency (n)	Percentage (%)	Statistical test
≤20	145	14.3%	χ² = 15.32, p = 0.002
21 to 30	79	7.8%	χ² = 8.12, p = 0.045
31 to 40	110	10.9%	F = 5.91, p < 0.01
41 to 50	219	21.6%	χ² = 12.65, p = 0.011
51 to 60	202	20.0%	χ² = 9.43, p = 0.039
61 to 70	156	15.4%	χ² = 7.56, p = 0.035
>70	101	10.0%	χ² = 5.78, p = 0.067

Majority of the inpatients were from the General Medicine department (n = 443), followed by General Surgery (n = 136), and the Nephrology department (n = 133). ANOVA revealed significant differences in the mean length of hospital stay across departments (F = 7.56, p < 0.001). Post-hoc analysis using Tukey’s honestly significant difference (HSD) indicated that patients in General Medicine stayed longer than those in Surgery (p < 0.05). Tukey’s HSD test is used to find which specific group means are different after finding an overall significant result in an ANOVA test. The chi-square test showed a significant association between department and antibiotic exposure (χ² = 34.29, p < 0.001), indicating that patients in General Medicine were more likely to receive multiple antibiotics. The distribution of patients across departments is presented in Table 2.

**Table 2:** Departmental distribution and antibiotic exposure

Department	Percentage (%) distribution	No. of admissions	No. of antibiotic exposure					
			0	1	2	3	4	≥5
General Medicine	43.8	443	51	200	107	56	15	14
General Surgery	13.4	136	1	70	44	18	3	0
Nephrology	13.1	133	4	66	35	18	5	5
Gynecology & Obstetrics	9.0	91	5	47	26	12	1	0
Pediatrics	5.0	51	4	17	17	8	4	1
Neurosurgery	3.4	34	2	24	5	2	0	1
Urology	3.1	31	0	28	2	1	0	0
Neonatology	2.4	24	2	0	20	1	1	0
Cardiology	1.5	15	6	9	0	0	0	0
Medical Oncology	1.5	15	6	3	1	3	0	2
Orthopedics	1.2	12	0	4	4	3	1	0
Otorhinolaryngology	0.8	8	0	6	2	0	0	0
Plastic Surgery	0.7	7	0	5	2	0	0	0
Gastroenterology	0.6	6	2	4	0	0	0	0
Oral Maxillofacial Surgery	0.5	5	0	1	4	0	0	0
Ophthalmology	0.1	1	0	0	0	0	0	1
	100	1012	83	484	269	122	30	24

**Figure 2:**
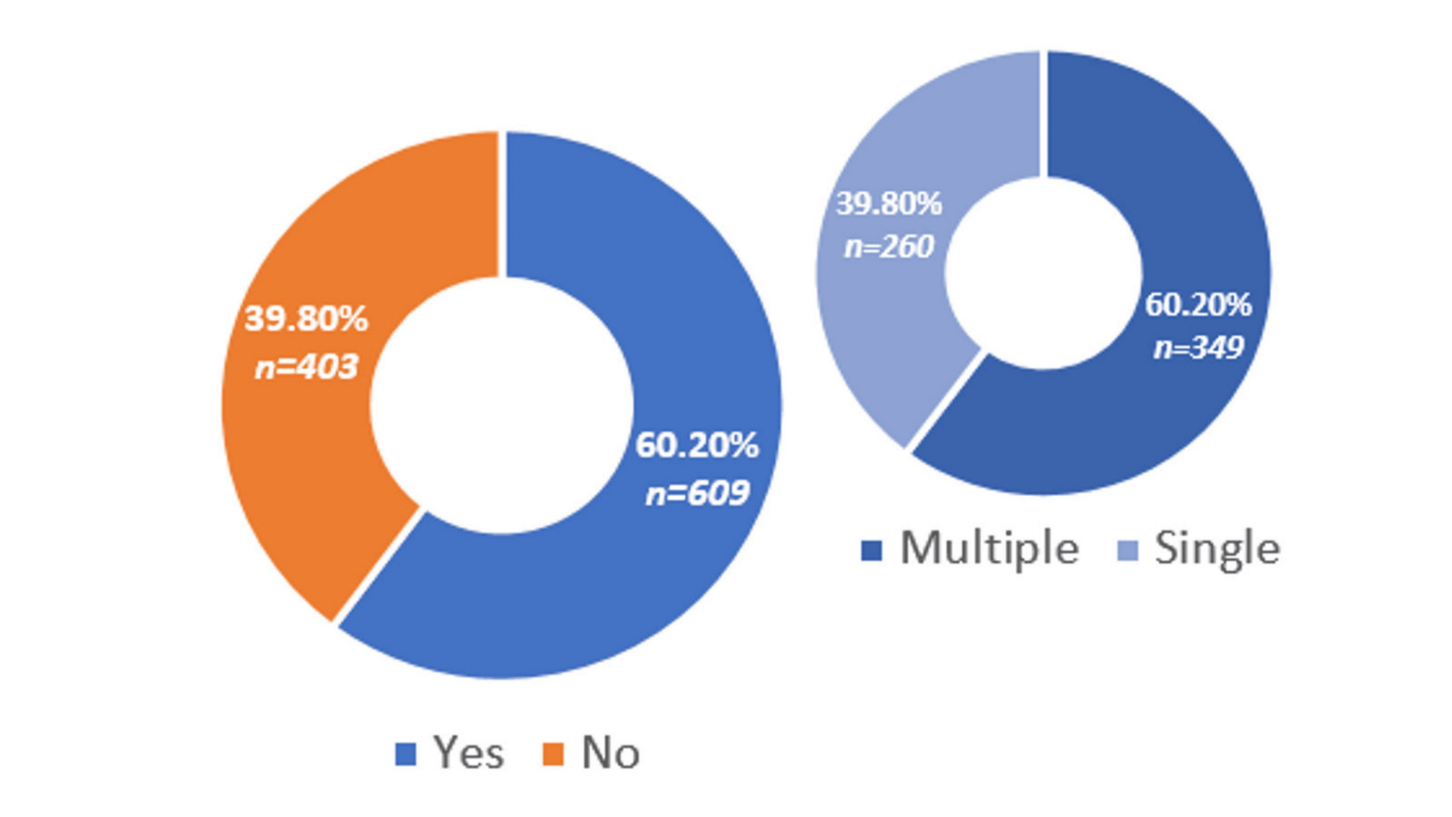
Comorbids conditions

**Figure 3:**
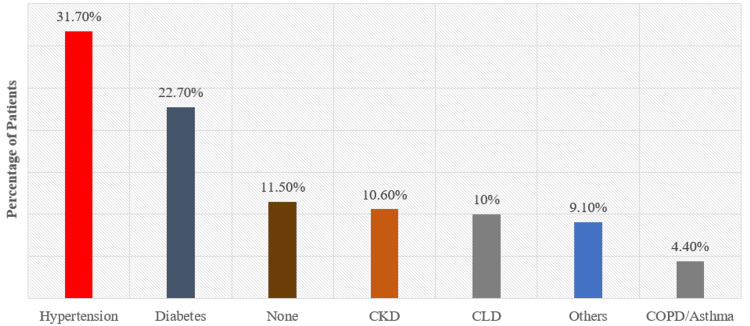
Spectrum of comorbid illnesses CKD: Chronic kidney disease; CLD: chronic liver disease; COPD: chronic obstructive pulmonary disease

A total of 60% (n = 609) of the patients had one or more comorbid conditions. A Chi-square test showed a significant association between comorbidities and antibiotic exposure (χ² = 21.78, p < 0.001), indicating that patients with two or more comorbidities were more likely to receive multiple antibiotics. Further analysis using Cox proportional hazards regression demonstrated that comorbidities were a significant predictor of ICU mortality (HR = 3.12, 95% CI: 1.58-4.76, p < 0.01). The comorbid conditions are shown in Figure 2 and Figure 3.

**Figure 4:**
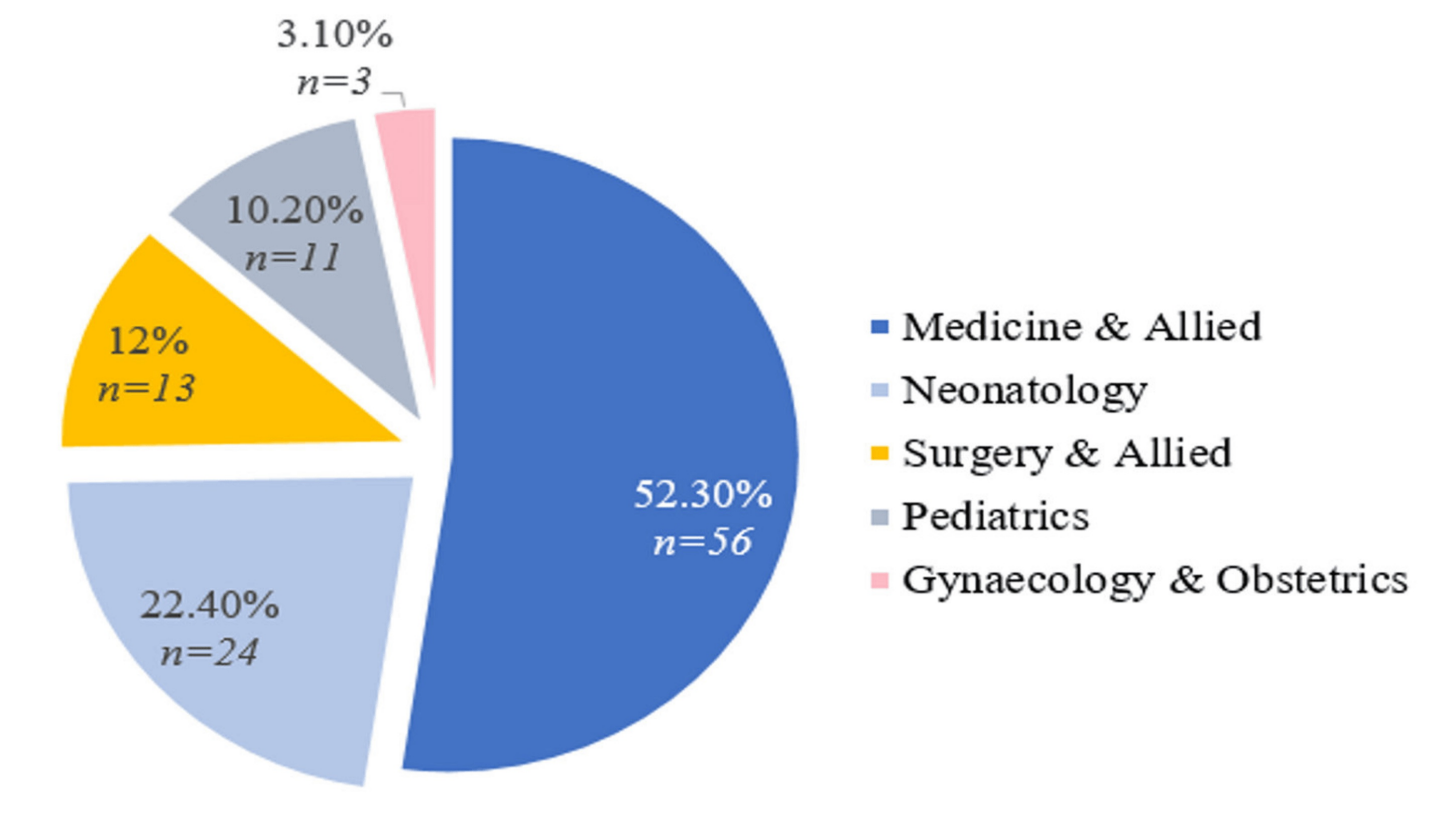
Departmental distribution of intensive care unit admissions

The number of intensive care unit (ICU) admissions was 107: adult ICU, n = 72; neonatal ICU, n = 24; and pediatric ICU, n = 11. Most ICUs were from the medical department followed by Neonatology and Surgery. Figure 4 shows the departmental distribution of intensive unit hospitalization.

**Figure 5:**
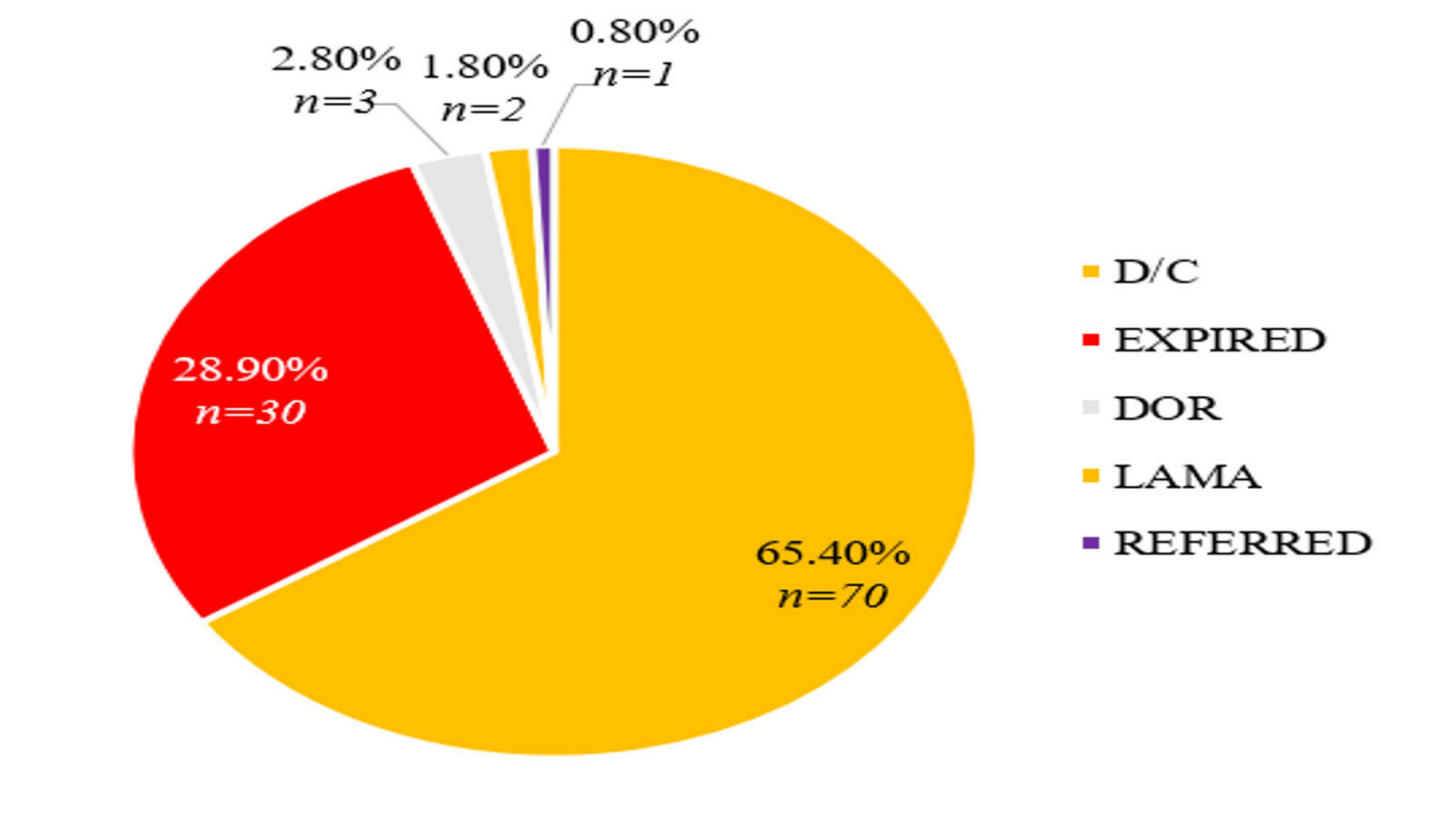
Outcome of ICU adimssion D/C: discharge; DOR: discharge on request; LAMA: left against medical advice

**Figure 6:**
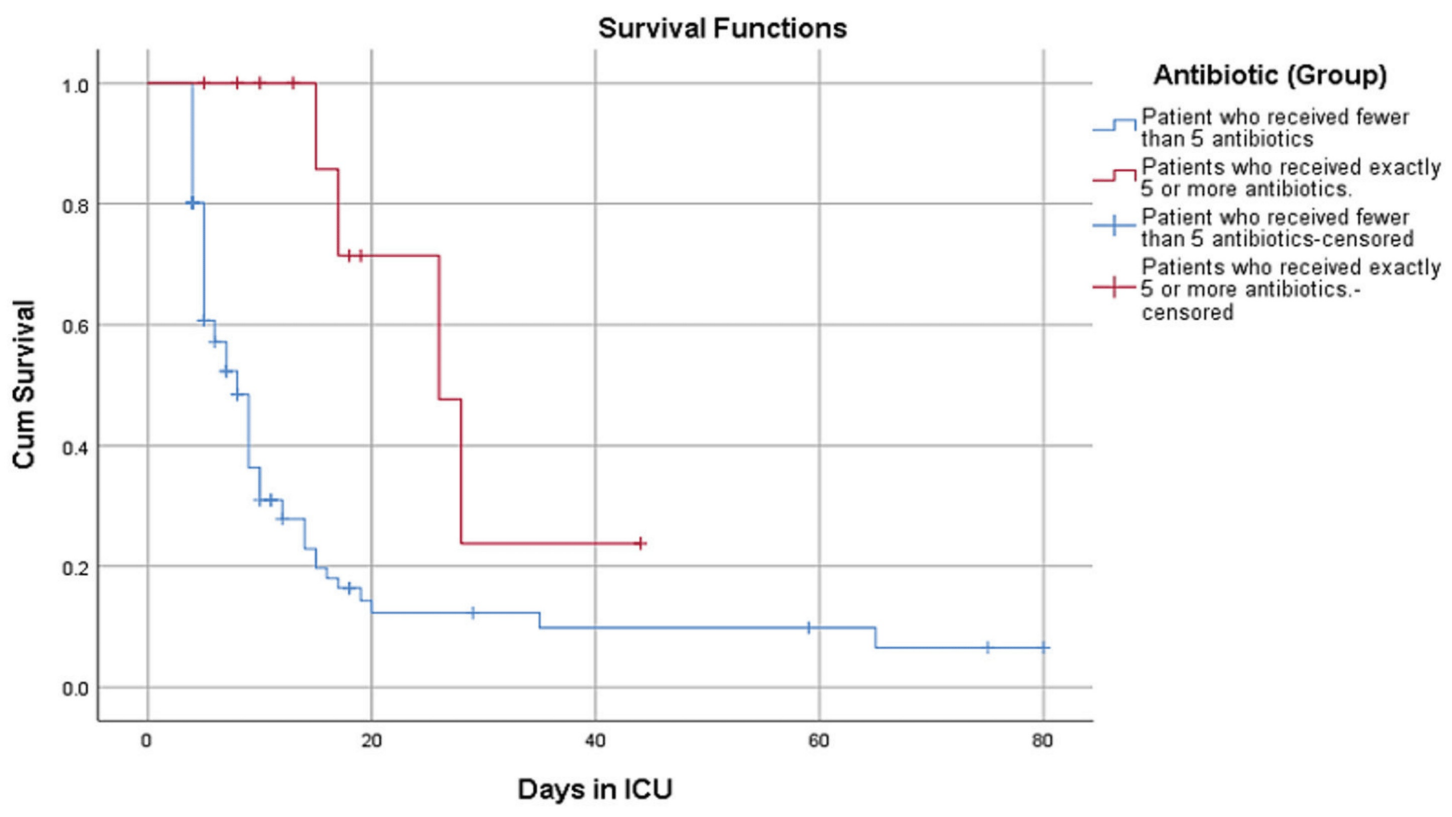
Kaplan-Meier survival analysis of ICU patients ICU: Intensive care unit

In the ICU, the rate of expiry was 28.9% (Figure 5) of the admitted patients, with higher mortality observed among patients receiving five or more antibiotics. The Kaplan-Meier survival analysis (Figure 6) demonstrates a clear inverse relationship between the number of antibiotics prescribed and ICU survival. Patients receiving five or more antibiotics had significantly lower survival rates (Log-rank test, p = 0.02), indicating that overprescription in critical settings can be detrimental to patient outcomes. This finding supports the hypothesis that excessive antibiotic use, particularly in ICU settings, leads to poorer prognosis. Cox proportional hazards regression identified comorbidities (two or more) as a significant predictor of ICU mortality (HR = 3.12, 95% CI: 1.58-4.76, p < 0.01). A Pearson’s correlation analysis was conducted to assess the relationship between the length of hospitalization and the number of antibiotics prescribed. A moderate positive correlation (r = 0.42, p < 0.001) suggests that patients who received more antibiotics tended to stay in the hospital longer.

**Figure 7:**
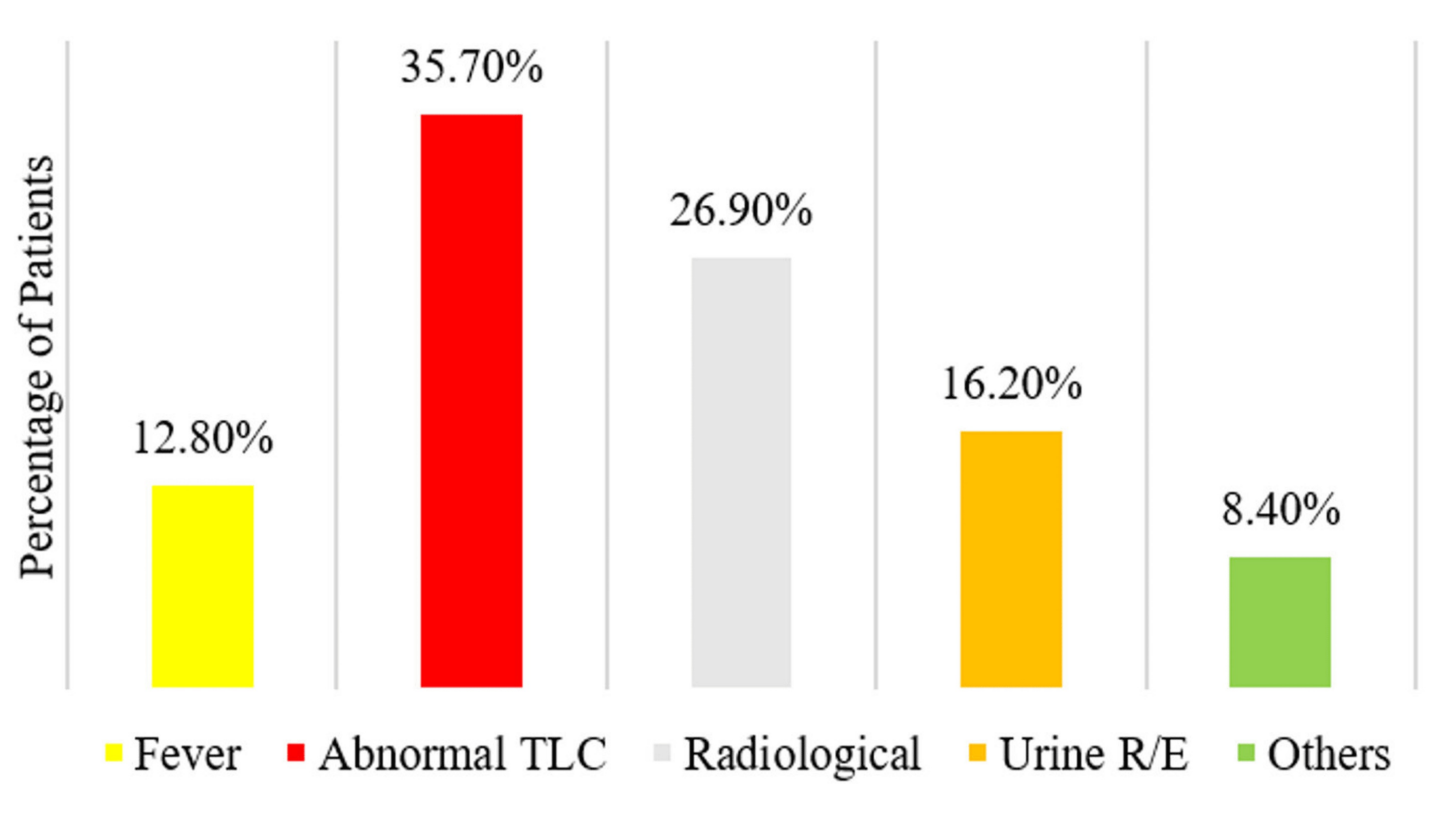
Parameters of evidence of infection (EoI) Urine R/E: Routine examination

The EoI was 68.7% (n = 695). Out of these abnormal total leukocyte count (TLC) was the most common finding. Figure 7 shows the overall distribution among the defined parameters of EoI.

A total of 91.8% of the patients (n = 929) were exposed to one or more antibiotics during their stay. Among these, 68.7% (n = 695) had one or more EoI. A total of 25.2% of the patients received antibiotics without clear EoI. A one-sample t-test comparing the mean exposure against a hypothetical value of 1 (representing minimal antibiotic use) was significant (p < 0.05), suggesting a higher-than-expected usage of antibiotics in this population.

The mean number of antibiotics prescribed per patient was 1.62 (±1.08 SD), indicating moderate variability in the number of antibiotics administered. A Chi-square test indicated a significant association between the presence of EoI and antibiotic exposure (χ² = 45.12, p < 0.001), with 68.7% (n = 695) of the patients with EoI receiving antibiotics. Multivariate logistic regression revealed that patients with EoI were 4.5 times more likely to be prescribed antibiotics compared to those without EoI (OR: 4.50, 95% CI: 2.90-6.80, p < 0.001). ANOVA revealed significant department-wise differences in antibiotic exposure (F = 5.21, p < 0.01), with ICU patients receiving more antibiotics compared to other departments. The detailed breakdown of antibiotic exposure is shown in Table 3.

**Table 3:** Antibiotic(s) exposure Statistical test: χ² = Chi-square test; F = ANOVA test Significance level: p < 0.05

Number of antibiotic(s)	Frequency (n)	Valid percentage (%)	Statistical test
1 Antibiotic	484	47.8%	χ² = 10.24, p = 0.001
2 Antibiotics	269	26.6%	χ² = 8.32, p = 0.004
3-4 Antibiotics	152	16.3%	F = 6.54, p < 0.01
≥5 Antibiotics	24	2.7%	F = 4.12, p = 0.02

The most commonly used antibiotics were third-generation cephalosporin "ceftriaxone" (n = 346) followed by the combination of "cefoperazone and sulbactam" (n = 235). The Chi-square test showed a significant association between the department and type of antibiotic prescribed (χ2 = 34.29, p < 0.001), with cefoperazone-sulbactam being more frequently prescribed in Medicine and Allied departments, and ceftriaxone being more common in other departments. Logistic regression indicated that patients admitted to surgical departments were twice as likely to receive a combination antibiotic therapy compared to those in medical departments (OR: 2.15, 95% CI: 1.32-3.49, p < 0.01). An ANOVA test confirmed statistically significant differences in antibiotic prescribing between departments (p < 0.001), particularly between Pediatrics and the other departments (Tukey’s HSD, p < 0.01). Table 4 shows the most commonly used antibiotics among the departments.

**Table 4:** Commonly prescribed antibiotics Statistical test: χ² = Chi-square test; F = ANOVA test Significance level: p < 0.05

Department	Most commonly used antibiotics	Statistical test
Medicine & Allied	Cefoperazone & sulbactam > ceftriaxone	χ² = 12.34, p < 0.01
Surgery & Allied	Ceftriaxone > cefoperazone & sulbactam	χ² = 9.21, p = 0.02
Obstetrics & Gynecology	Ceftriaxone > metronidazole > ciprofloxacin	χ² = 7.65, p = 0.015
Pediatrics	Ceftriaxone > amikacin > meropenem	χ² = 5.48, p = 0.023

**Figure 8:**
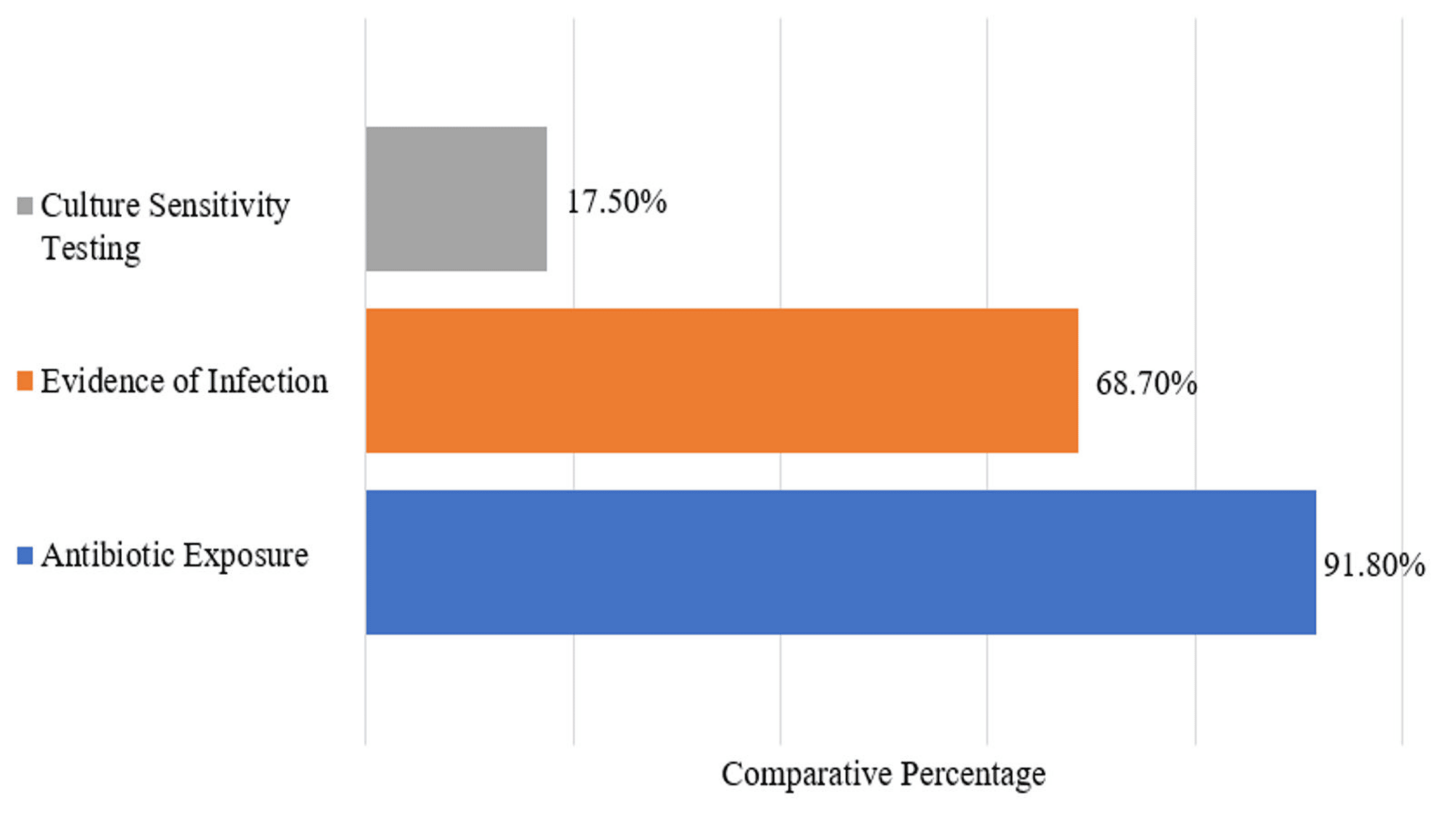
Comparative analysis of CS testing, EoI, and antibiotic exposure CS: Culture Sensitivity; EoI: evidence of infection

Despite the extensive use of antibiotics, only 17.5% (n = 163) patients underwent CS testing, some of whom had multiple tests, resulting in a total of 176 CS reports. Among 176 CS reports, 38 (21.6%) yielded positive cultures at admission and 32 (18.2%) during hospital stay. Figure 8 shows a comparative picture of antibiotic exposure, infection evidence, and CS testing.

A McNemar’s test was conducted to compare the (CS results at the time of admission with those taken during the hospital stay. The results indicated no significant difference between the two time points (p = 0.35), suggesting that the timing of the test did not significantly affect the likelihood of obtaining a positive culture result. Logistic regression analysis indicated slightly higher odds of a positive culture at admission (OR = 1.18), though this was not statistically significant (p = 0.35). The Chi-square test demonstrated a significant association between CS testing and antibiotic switching (χ² = 18.64, p < 0.001). Patients with positive culture results were 4.2 times more likely to have their antibiotic regimen adjusted (OR: 4.22, 95% CI: 2.75-6.31, p < 0.001). Characteristics of CS tests are described in Table 4.

**Table 5:** Culture sensitivity testing characteristics MRSA: Methicillin-resistant *Staphylococcus aureus*

Test type	Total	Positive	Negative	On admission	During admission	Antibiotic changed	Antibiotic not changed	Most common organism
Urine	79	34	45	41	38	21	24	*E. coli* (50%), *Enterococcus *(14.7%)
Blood	48	6	42	16	32	5	1	Staph/Strep (33.3%)
Pus	29	21	8	12	17	17	4	*E. coli *(28.5%), MRSA (23.8%)
Other fluids	20	9	11	5	15	7	2	*Candida albicans* (44.5%)

## Discussion

The results of this study confirm that inappropriate antibiotic prescribing remains a significant concern in Pakistani hospitals, with 30% of patients receiving antibiotics without documented EoI. This trend is consistent with the findings from Steinke et al. [11], who reported a 34% rate of inappropriate antibiotic prescriptions in general practice settings, and Arnold et al. [12], who identified a 28% rate of inappropriate use in pediatric respiratory infections. The high empirical use of antibiotics without CS testing further exacerbates the issue. In resource-limited settings like Pakistan, where diagnostic capacities are constrained, this practice contributes to the growing burden of MDR pathogens.

The absence of culture testing in 82.5% of cases in this study underscores the pressing need for enhanced diagnostic infrastructure. While empirical therapy may be necessary in acute settings, the systematic neglect of CS testing increases the risk of treatment failure, prolonged hospital stays, and heightened mortality, especially in critical care units. This finding aligns with global observations of inappropriate antibiotic use in resource-limited settings [7,19,20]. Our findings are aligned with those of Wolkewitz et al. [21], who reported a similar association between broad-spectrum antibiotic use and increased mortality in ICU patients.

Furthermore, a significant portion of patients received antibiotics during hospitalization, but CS testing was performed in only a minority of cases. This practice exposes patients to unnecessary polyantibiotic therapy and potentially increases the length of hospitalization and the risk of treatment failure, particularly when antibiotics are prescribed without definitive evidence of infection. The data reveals a clear trend of overprescription, with 30% of patients receiving antibiotics without documented EoI. This finding is consistent with Wolkewitz et al. [21] and Rhee et al. [22] studies, where inappropriate antibiotic prescriptions were driven by clinician concerns about patient safety, lack of diagnostic capabilities, and time pressures.

Looking beyond the hospital setting, the implications of our study resonate with broader national and international AMR strategies. The WHO has emphasized the critical need for multisectoral action, including public health initiatives and strengthened hospital protocols, to reduce irrational antibiotic use. The Pakistan National Action Plan on AMR 2017 outlines a multisectoral approach focusing on strengthening laboratory capacity, surveillance, and implementing ASPs in healthcare facilities [7]. However, its implementation has faced challenges due to limited resources and infrastructure.

To mitigate these issues, ASPs should integrate mandatory CS testing protocols and emphasize clinician education on the rational use of antibiotics. Evidence suggests that pharmacist-led interventions and stricter hospital guidelines can significantly improve antibiotic use in healthcare settings [14,15]). Incorporating these strategies into the national policy would enhance compliance and help curtail the rise of MDR pathogens in Pakistan.

Limitations of the study

This study has several limitations. First, the retrospective nature of the study limits our ability to establish causality. Second, the lack of randomization and a control group restricts comparisons of antibiotic prescribing practices. Additionally, only a small percentage of patients underwent CS testing, potentially affecting the generalizability of the findings regarding the appropriateness of antibiotic use. Future research should incorporate prospective designs with randomized control groups and broader diagnostic protocols to assess antibiotic stewardship more comprehensively.

## Conclusions

This study identified that 91.8% of patients received antibiotics, with 30% being prescribed without documented EoI. It was also noted that only 17.5% of patients underwent CS testing, raising concerns about empirical therapy. This study provides a comprehensive overview of antibiotic prescribing practices in Pakistani hospitals and underscores the urgent need for policies that address the irrational use of antibiotics, which significantly contributes to AMR. The findings highlight the critical need for targeted interventions, such as the implementation of ASPs that prioritize mandatory CS testing and clinician education. These programs are essential to ensure rational antibiotic use, reduce hospital costs, and minimize the spread of multidrug-resistant pathogens.

Addressing these challenges requires both institutional and national policy efforts to optimize diagnostic protocols and antibiotic stewardship. By integrating these strategies, healthcare systems can better combat the growing threat of AMR and improve patient outcomes. The results of this study have important implications for public health and should inform the development of tailored programs that emphasize effective antibiotic supervision, diagnostic testing, and clinician training.
